# The Relations of the Board of Dental Examiners to the Profession

**Published:** 1903-02-15

**Authors:** H. C. Brown

**Affiliations:** Columbus, O.


### THE RELATIONS OF THE BOARD OF DENTAL EXAMINERS TO THE PROFESSION.

BY H. C. BROWN, D.D.S., COLUMBUS, O.

Read before the Ohio State Dental Society, December 3, 1902.

In presenting this paper I desire to state that I was reluctant in accepting this invitation inasmuch as my experience as an examiner has only been of short duration. Nevertheless I will give my views and incorporate a report of the work of our board.

Our present law regulating the practice of dentistry was passed April 29th, 1902, after much delay, with quite a number of changes from the bill as originally prepared, but an error was discovered, making it necessary to amend section 4404, which was done May 10th, 1902, giving us a much improved law over the one in force for the past ten years, which was very lax in its provisions, thereby permitting Ohio to be the "dumping ground," so to speak, of adjoining States, as it was only necessary that an applicant possess a diploma from some legally chartered dental colleges and pay a fee of $2.00 to secure a certificate entitling him to practice in Ohio; while our present law provides that any one who is a legal resident of the State and has been a student of dentistry, under a preceptor for one year prior to the passage of this act, shall be permitted to take the examination during the years 1902 and 1903; and that we register those who have been proprietors of offices in the State continuously since January 1st, 1893; also that we register all graduates of dental collegesof this State up to and including the June , 1905, session of the board, all other applicants being required to present a diploma and pass the examination before certificates of registration are issued them. Therefore, this eventually becomes a very strict law, and after June, 1905, the dental profession of the State will have legislation that is uniform in its provisions, and will compare favorably with other States having high standard requirements.

In the reports of the various State Examiners at the National Association of Dental Examiners at Niagara Falls, last July, I was surprised to find that nearly everyone admitted more or less weakness in provision of their respective State laws. However, I may say that all are aggressive and do not permit illegal practice.

It is not equitable to those who are inclined to be legal practitioners and comply with every requirement of any new legislation, thereby assisting materially in the elevation of our profession, and at the same time protecting the public and themselves, that others be permitted to practice without strictly complying with the same requirements, whether or not it be their inclination.

I am firmly convinced that there is much illegal practicing done in Ohio, and possible more than in any other State.

This is due to our inability to secure proper legislation in the past. A person who has practiced for a long time without interference, reasons that if it has been unnecessary to register before why go to that trouble and expense now?

I am confident that there are a number of dentists practicing illegally, who are graduates of the different dental colleges of this State and therefore are eligible to register upon proper application and fee of ten dollars, while perhaps equally as many are graduates of other colleges, and their neglect or ignorance will cost them $18.00 additional fee and the expense, to say nothing of the unpleasant features of taking the examination.

There is another class of illegal practitioners who have been students of preceptors or are under graduates. They are eligible to examination during the years 1902 and 1903, if they are legal residents of Ohio, and have been students under preceptors for one year prior to passage of this act.

I regret that one important feature, although it may seem insignificant, of the former and present law has been disregarded by some of our best men of the profession. I refer to the clause which provides that the registration certificates shall be placed in a conspicuous place in the office. This is one of the reasons why it is difficult to ascertain whether or not a practitioner is registered, without referring to the books for information. A diploma may be where it can be seen, but it is the certificate that makes the person a legal practitioner. Do not consider that I desire to detract from the importance of a diploma, nor criticise you for having it where it can be seen, if not read; however, I do consider it quite important that your certificate be placed as the law provides, and by so doing you will not only meet the requirements of our law, but will assist in establishing a uniformity that should exist, making it the more difficult for anyone to practice illegally. I know of a number of registered dentists who, in their endeavor to take especial care of their certificates, issued some years ago, were particular to put them away in some safe place and have never seen them since, and perhaps never will.

Our board will issues duplicates to such persons, upon proper application and affidavit that certificate has been lost or destroyed, for a fee of $10.00.

I deem it quite unnecessary for me to say that the relations between the profession and the board of examiners should be harmonious, and that the success of any examining board will depend, first, upon the examiners themselves, secondly, upon the law under which they are appointed, and last but not least, upon the hearty co-operation of the profession. It is the public as well as the profession we are supposed to protect. Although the public may not appreciate the interest shown and consider it purely mercenary, I believe the time is not far distant when we may hope to realize that our efforts have been appreciated, and that it will be manifested in raising the educational requirements. This law was not enacted for those practicing the profession and at the expense of the public.

The profession should be on the lookout to secure such legislation from time to time as is needed to meet the changing requirements. In procuring legislation there should be more interest displayed by the entire profession than has shown itself in the last few years, when efforts have been made to secure needed legislation. I desire here to state that some criticisms may be offered to certain provisions that are embodied in our present law. If everyone knew of the time and energy which Dr. Geo. H. Wilson, as secretary of the legislative committee of the State Society, gave to making this law fit the conditions existing when certain changes were proposed, I assure you that he would think twice before he spoke critically, and have reason to regret that perhaps he did not do his entire duty. In seeking legislation it should be remembered that it is quite impossible to thoroughly satisfy the profession of the State as a body, the State Society, or any local society, or even to secure the hearty approbation of any Examining Board that should be appointed. Compromises should be made and all should gladly assist in securing such changes as are deemed necessary to meet the advanced requirements.

The Board of Examiners is executive and judicial. Executive inasmuch as it is its duty to see that the provisions of the law are enforced. It is given power to make such rules and regulations for carrying out the provisions of the law and conducting the affairs of the board as may be needed It has been decided by Attorney Generals and the Supreme Courts of different States that it has judicial functions. Two recent Supreme Court decisions were rendered in Wisconsin and Illinois, respectively. In an exhaustive review the Supreme Court of Wisconsin sustains the Board of Examiners in every position and among other things says: "The State Board of Dental Examiners, proceeding reasonably, is the sole tribunal under the statute to determine the questions of fact to be solved, preceding the licensing of a person to practice the profession of dentistry in this State."

In a very able paper regarding this decision, read before the American Medical Society, by Dr. Charles C. Chittenden, a member of the Wisconsin Board of Dental Examiners and chairman of the Committee on Colleges of the National Association of Dental Examiners, he says: "The deduction of your essayist from all this is that the question of unifaction of dental laws and interstate reciprocity of license, together with unifaction of dental educational standards on any reasonably high plane 'within the bounds of common sense,' is in the hands and entirely within the province and power of the State Boards, provided they will act in concert in carrying out the high duties imposed upon them as the dental police officers of their several States, and this, too, without the enactment of any new dental legislation whatever. So it will seem that the statutes in every state in the Union grant its State Board all the judicial power necessary to join in establishing absolute National standards of dental educational requirements. There is absolutely nothing in all this proposed unifaction of standards, etc., by the boards that can react in any but a beneficial manner upon the schools themselves. The element of commercial competition will be eliminated where it exists, and in its stead the spirit of emulation will be greatly stimulated."

Our law provides that "upon a unanimous vote of the Board we may excuse from examination an applicant holding a license to practice in some state, requiring a diploma and examination, upon the payment of examination fee." For the present we do not recognize such certificates, yet we appreciate the justness of such recognition and stand willing at any time to lend our efforts toward establishing high standard interchangeable relations, but after an unsuccessful effort on our part to install such relations, we do not consider that we would be treating the profession of Ohio fairly, nor aiding in the unifaction of all State dental laws. I believe, after 1905, the time when our law becomes uniform, that then it will be possible to have reciprocal relations with some, at least, of the leading high standard States.

The question of an advisable method to pursue in regard to such as are practicing illegally and how far the Board may be justified in going to discover all unregistered practitioners with a view of enforcing the provisions of the law, is a more or less perplexing one. It is the "duty of the prosecuting attorney of each county to prosecute every case in final judgment whenever his attention shall be called to a violation of the provisions of this law;" but the difficulty arises from the fact that as a board we have no knowledge of the legal standing of the profession in this state, other than as we can obtain it from the list of those that are registered. You can have no positive knowledge as to whether the dentists of your community are all registered, unless you write the secretary of the Board for such information. I will state that, as secretary, I have received a great many such inquiries and have always answered them promptly, giving the information asked, and as long as I remain secretary, I shall give that and other business as prompt attention as possible.

To obtain an accurate list of dentists practicing in this State, I think a prominent member of the profession in each county should be requested to furnish the Board with the names of all those practicing in his county. This would be an accurate way of obtaining the information, especially from the counties of moderate population. And as for cities, the four larger ones have representation on the Board, and each could report for his city.

I realize this will require additional work for myself, as secretary of the Board, but if it is the only way to obtain this information, I shall willingly do my part. Personally, I do not consider there is any occasion for anyone practicing in this state without being registered, and I believe it reasonable to hope for such a result, before this society meets again; provided, of course, that our Board has the earnest co-operation of those members of the profession who are registered.

It is impossible for us as a profession, or a Board to control or regulate the methods of conducting a practice, but while our law is very explicit regarding assistants, we can require that all persons employed in offices and performing dental work, shall be registered. This does not apply to the unethical any more than to those conducting an ethical practice. I trust that all who employ assistants will recognize the importance of this provision and see that their assistants are legal practitioners, thereby assisting in eliminating this troublesome question as its tendency is to evade this provision of the law.

In making a report of the work done by the Board, I will state that the present board was appointed June 5th, and organized June 9th, 1902. We have held two examinations, as provided for by law, beginning June 24th and Nov. 25th, respectively. Seventeen applicants took the first examination, fourteen of them passing; at the examination last week we had fifteen applicants and one for re-examination who had failed last June, but as yet the results are unknown.

Most of the applicants have been graduates of colleges outside of Ohio, and they represent some of the leading schools of the country; for instance, of the fifteen applicants last week, six of them were graduates from the University of Michigan.

Our Board recognizes the full import of the practical part of an examination, and each applicant has an opportunity to present evidence of ability in the various clinical requirements that we demand.

The following is sent to each applicant: "Applicants for examination are required to bring instruments, engine, plate punch, files and impression trays and the following materials: gold and silver plate and solder for same, alloy, cement, rubber dam, facings and gum section teeth.

"They will be required to prepare a cavity and insert gold filling, and if requested, fill a tooth with alloy or cement; take plaster impressions and secure model therefrom; grind gum section teeth; back facing, invest and solder with either silver or gold.

The dental department of the Ohio Medical University has kindly given us the privilege of using their college and equipment in order to make this feature of our examination as practical as possible and if it was not for this opportunity, it would be impossible for us to accomplish the same results in this particular part of our examination. Our Board endeavors to be practical, not technical, and I think our clinical requirements are equal, if not superior, to those of any State. In anticipation of changes in the law, a great many graduated of colleges from other states were desirous of registering under the provisions of the former law, but some graduated a day or so too late. On advice of the Attorney General, we registered fifty-five, whose applications were filed legally with former board before the passage of present law, at the former registration fee of two dollars ($2). We returned fees to perhaps fifty (50), whose applications were not received in time to permit them to be registered under former law.

Besides the fifty-five (55) above mentioned, we have registered one-hundred and five, making a total of one-hundred and sixty (160), which makes a grand total of 3,235 who have registered in this state since the passage of the former law in 1892. However, a great many of those who have registered have not, and perhaps never will, practice in this State, as they only took advantage of lax condition of law and to prepare for future emergencies.

In closing, I will state that we require a clinical grade of 80 percent and theoretical of 75 percent, and that we only know an applicant by the number assigned at the beginning of examination.

